# The Effect of SMS Reminders on Adherence in a Self-Guided Internet-Delivered Intervention for Adults With ADHD

**DOI:** 10.3389/fdgth.2022.821031

**Published:** 2022-05-16

**Authors:** Emilie S. Nordby, Rolf Gjestad, Robin M. F. Kenter, Frode Guribye, Suresh K. Mukhiya, Astri J. Lundervold, Tine Nordgreen

**Affiliations:** ^1^Division of Psychiatry, Haukeland University Hospital, Bergen, Norway; ^2^Department of Biological and Medical Psychology, Faculty of Psychology, University of Bergen, Bergen, Norway; ^3^Center for Crisis Psychology, Faculty of Psychology, University of Bergen, Bergen, Norway; ^4^Research Department, Division of Mental Health, Haukeland University Hospital, Bergen, Norway; ^5^Centre for Research and Education in Forensic Psychiatry, Haukeland University Hospital, Bergen, Norway; ^6^Department of Clinical Psychology, Faculty of Psychology, University of Bergen, Bergen, Norway; ^7^Department of Information Science and Media Studies, University of Bergen, Bergen, Norway; ^8^Department of Computer Science, Electrical Engineering, and Mathematical Sciences, Western Norway University of Applied Sciences, Bergen, Norway; ^9^Department of Global Public Health and Primary Care, Faculty of Medicine, University of Bergen, Bergen, Norway

**Keywords:** Attention-Deficit Hyperactivity Disorder (ADHD), Internet-delivered interventions, adherence, reminders, non-pharmacological treatment, SMS

## Abstract

**Background:**

Self-guided Internet-delivered interventions may serve as an accessible and flexible non-pharmacological treatment supplement for adults with ADHD. However, these interventions are challenged by low adherence.

**Objective:**

To examine whether weekly SMS reminders improve adherence to a self-guided Internet-delivered intervention for adults with ADHD.

**Method:**

The study used a multiple randomized trial design where the participants who had not completed their weekly module within 2 days were randomized to either receive or not receive an SMS reminder. The primary outcome was adherence, defined as module completion, logins, time spent on intervention, and self-reported practice of coping strategies.

**Results:**

A total of 109 adults with a self-reported ADHD diagnosis were included in the study. The results showed that SMS reminders were associated with an increased likelihood of login within 48 h during the second module of the intervention, but not for the remaining modules. Moreover, receiving an SMS reminder was associated spending more time on the modules and faster login time in module three and five, specifically. However, the overall results did not show an effect of SMS reminders on module completion, number of logins or practice of coping strategies.

**Conclusion:**

The results showed that SMS reminders do not improve number of logins, module completion rates or practice of coping strategies, but they may lead to faster login time and more time spent on the modules. To utilize the potential of self-guided Internet-delivered intervention in making non-pharmacological accessible for adults with ADHD, new methods to facilitate meaningful engagement should be developed and tested.

**Trial Registration:**

ClinicalTrials.gov NCT04511169.

## Introduction

Attention-Deficit Hyperactivity Disorder (ADHD) is a common neurodevelopmental disorder. The majority of childhood cases still experience impairing symptoms as adults and the prevalence in the adult population is estimated to be ~2.5% (1–3). The core ADHD symptoms can be described within three main domains: inattention, hyperactivity, and impulsivity (4), but other domains, such as emotion regulation (5, 6), sleep (7) self-esteem (8), and interpersonal relationships (9, 10) are also commonly affected. Taken together, the core symptoms of ADHD and the associated challenges lead to impaired functioning in many aspects of daily life (4, 10, 11).

Pharmacological interventions are the main choice of treatment for adults with ADHD (12), and have been found to alleviate core symptoms of ADHD in a safe and effective manner (13). However, many adults with ADHD also seek supplementary non-pharmacological treatments due to residual impairments or side-effects (14). There is emerging evidence for the efficacy of psychological interventions as a treatment option for adults with ADHD (15, 16). Still, most adults with ADHD do not have access to these treatments, which may be due to a lack of economic or therapist resources, geographical distance, or stigma to seeking treatment (17, 18). As such, there is a need to increase access to evidence-based, effective, and scalable interventions for this group of adults.

Self-guided Internet-delivered interventions may serve as a scalable treatment format that helps to increase access to evidence-based psychological treatment for adults with ADHD (19). However, a common challenge of self-guided Internet-delivered interventions is poor adherence (20, 21). Within Internet-delivered interventions, adherence is often defined as active usage of the intervention or usage of the intervention as intended, and it is commonly operationalized as the number of completed intervention elements or modules (22). Meta-analyses on self-guided Internet-delivered interventions for depression have found that 74% of the participants do not adhere to the treatment (23), and that 70% of the participants drop out before completing 75% of the modules (24). Moreover, among self-guided interventions distributed openly to the public, more than 90% drop out after two modules (20). Adherence to treatment may even be more challenging for adults with ADHD due to common symptoms like distractibility, forgetfulness, and problems with time management (12). A study that examined a self-guided Internet-delivered intervention for adults with ADHD found that 77% of the participants did not complete the intervention (25). This is a major challenge given that greater adherence is associated with better treatment outcomes in self-guided Internet-delivered interventions (21, 26). As such, several individuals accessing these interventions will most likely not receive a sufficient dose of the treatment to obtain clinical improvement (26).

Digital reminders could be a low-cost and scalable method to improve adherence in self-guided Internet-delivered interventions (27). In previous studies, digital reminders have been found to increase adherence rates in interventions for anxiety, depression, and sleep (20, 28). Short message service (SMS) could be a promising medium to deliver such reminders, given its high accessibility and low distribution cost. Another benefit of SMS reminders is that they are delivered momentarily and in people's natural environment (29). Moreover, SMS reminders can be of particular benefit to individuals who struggle with problems such as forgetfulness, where eliminating this obstacle to treatment adherence might also enhance self-efficacy (30). Among adults with ADHD, SMS reminders have been found to improve adherence to pharmacological treatment (31). As such, implementing SMS reminders in self-guided Internet-delivered interventions for adults with ADHD could potentially be an effective and feasible method to improve adherence rates.

Although implementation of SMS reminders is expected to improve adherence (20), as far as we know, no study has examined if SMS reminders improve adherence in self-guided Internet-delivered interventions for adults with ADHD. In addition, given that there is a risk for habituation with continuous SMS reminders, there is a need of knowledge concerning whether their effectiveness differs throughout the intervention. As self-guided Internet-delivered interventions are still a novel treatment format for this group of adults, our study expand current knowledge on how these interventions can be successfully delivered. The primary aim of the current study was to contribute to this task by examining whether weekly SMS reminders improve adherence to a self-guided Internet-delivered intervention for adults with ADHD.

## Methods

### Study Design

**Figure 1:**
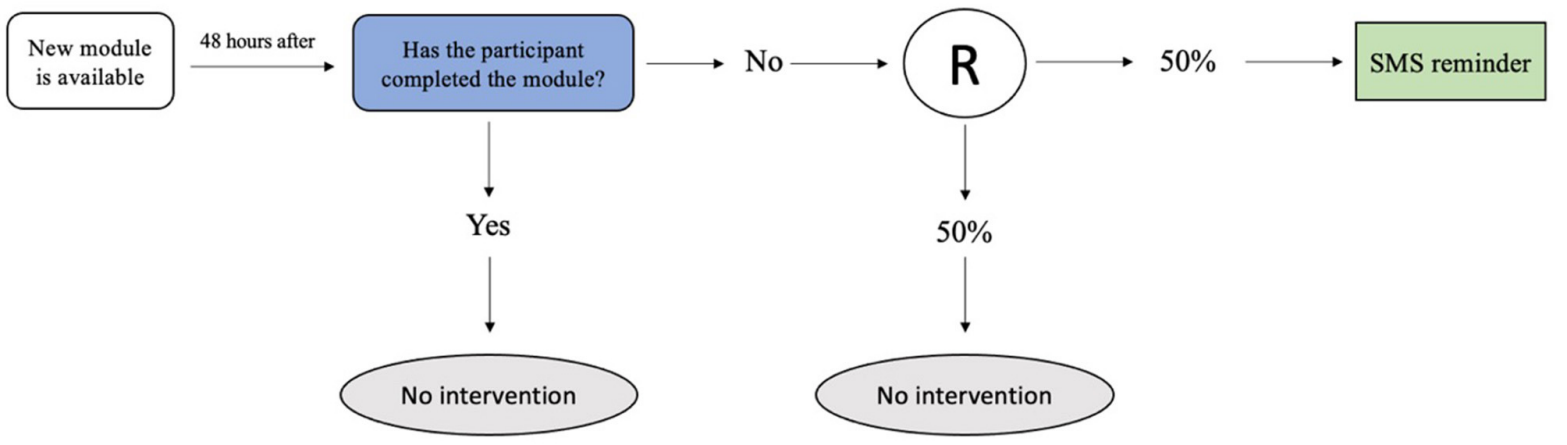
Study design.

The current study used a multiple randomized trial design (see Figure 1). The participants who did not complete their weekly module within 48 h after receiving access to the module, were randomized to either (a) receive an automated SMS reminder or (b) not receive a reminder. The remaining participants who had completed the module within 48 h were not included in the randomization. The randomization was repeated for each weekly module, with a total of six possible randomizations for each participant.

A multiple randomized trial design was chosen as it allows for investigation of time-specific effects of the SMS reminders, unlike traditional randomized-controlled trials, which only allows for assessment of the overall effect of receiving the SMS reminders (32). Given the risk of habituation with SMS reminders, we found it likely that there would be time-varying effects.

### Sample

Eligible participants were adults with a self-reported diagnosis of ADHD living in Norway. Participants were recruited through two approaches: (i) we contacted participants on an interest list from a previous advertisement; (ii) the Norwegian ADHD patient organization sent an email to its members informing them about the study and shared a post about the study on their official Facebook page. The data was collected between May 2020 and November 2020.

The inclusion criteria for the study were: (i) age 18 years or older; (ii) a self-reported diagnosis of ADHD; (iii) access to a computer or smartphone with Internet access, (iv) the ability to read and write the Norwegian language.

The exclusion criteria were: (i) severe mental illness, such as suicidality, psychosis, or substance abuse; (ii) ongoing psychological treatment for ADHD or another psychiatric disorder.

### Procedure

Interested participants were directed to a study website that contained information about the study and a pre-screening survey to evaluate eligibility. To be evaluated as eligible after the pre-screening, the participants had to confirm questions about the inclusion and exclusion criteria. Eligible participants were contacted for further evaluation in a phone interview with a clinical psychologist or psychiatric nurse. To be included the participants had to confirm the presence of an ADHD diagnosis and the time and place for the diagnostic assessment. The participants were also screened for depression, psychosis, bipolar disorder, and substance abuse using the MINI international neuropsychiatric interview (MINI) (33), and suicidality using item ten from the Montgomery and Åsberg Depression Rating Scale (MADRS) (34). Those who reported any of the aforementioned diagnoses were excluded. After the phone interview, the included participants got access to the intervention platform where they signed an informed consent form and completed the pre-intervention assessment. The participants were also asked to complete weekly questionnaires assessing inattention, a post-intervention assessment at the end of the intervention, and a follow-up assessment 3 months later. All participants received a gift card of 400 NOK upon completing the follow-up assessment.

### Intervention

The intervention was developed for adults with ADHD with a focus on core challenges associated with ADHD. The intervention applied an integrative framework using principles from Cognitive Behavioral Therapy, Goal Management Training and Dialectic Behavioral Therapy. A total of seven modules were included and the modules were released in a one-week interval, meaning that the participants were expected to use 1 week to complete one module. The modules included coping strategies, psychoeducation, exercises, lived-experience videos, and case vignettes [see (35, 36) for more information regarding the content and development of the intervention]. The participants were given access to the first module of the intervention after completing the pre-intervention assessment. All participants received an SMS when new content was available. The SMS stated that a new module was released and contained a web link to the intervention portal. For the post- and follow-up assessment, the participants received two additional SMS reminders and thereby one phone call if they had not completed the assessments. A clinical psychologist or a psychiatric nurse monitored the participants' responses on questionnaires and open text fields and were ready to contact any participant who indicated adverse effects or reported suicidal ideation during the trial.

We have previously examined the acceptability, credibility, and preliminary clinical effects of the first three modules of the current intervention in a small feasibility trial including 13 adults with ADHD (37). Overall, the participants reported good credibility, satisfaction, and positive preliminary clinical effects, but the adherence was suboptimal, with about 50% of the participants completing these first three modules (37). We made considerable adjustments to the design of the intervention platform based on information from this feasibility trial.

#### Randomized SMS Reminders

The participants who had not completed the new module within 48 h were randomized to receive one extra SMS reminder or no reminder. This SMS reminder stated that the participant had not completed their weekly module and encouraged them to continue with the program. The SMS also included a web link to the intervention portal where the participants could access the module. All randomized SMS reminders were sent in the afternoon, at ~5 p.m.

### Outcome Measures

#### Module Completion

A module was automatically registered as completed if the participant had read through all pages of the module. The number of modules completed could range from 0 to 7. The participants who completed at least five out seven modules of the intervention were defined as treatment completers. To examine the proximal effect of the SMS reminder we also examined whether the participant had completed their module within 1 week after the module was released.

#### Logins

We assessed both number of logins to the intervention portal, as well as the time of login. To examine the proximal effect of the SMS reminder we examined login within 48 h of the SMS reminder. Number of logins and time of logins were automatically registered in the intervention portal.

#### Minutes Spent Online

Time spent online refers to how many minutes the participant spent online in the intervention. Both minutes spent on the program in total and minutes spent on each module were registered.

#### Use of Coping Strategies

At the post-assessment, the participants were asked how many days a week they spent practicing the coping techniques presented in the intervention. The participants could choose from the options: “Never” (1); “Less than once a week”; (2); “1 or 2 days a week” (3); “3 or 4 days a week” (4); “5 or 6 days a week” (5); or “Daily” (6).

### Statistical Analysis

Descriptive statistics, with mean, standard deviation, skewness, and kurtosis, were analyzed with IBM SPSS (38). SPSS was also used for cross-tabulations of the SMS reminder and module completion within a week and login within 48 h after the SMS reminder on the sub-sample within each module that were included in the randomization. The relationships were tested with Chi-square test. The program Mplus 8.7 was used for the remaining analyses (39). In order to use all available information, the randomized condition of giving SMS or not was represented with two contrast codes (Helmert contrasts H1 and H2) (40). The H1 captured being in the randomized group (H1 = 1) or in the group not in need for randomization (H1 = 0) within each module. The effects of SMS within modules were captured by the H2 code (SMS = 0.5, no SMS = −0.5, and the group not having to be randomized = 0). A procedure of several analyses was run for each outcome variable over the weeks 2–6. Unfortunately, the first week of the study had to be excluded from the analyses due a technical error that triggered the SMS reminders after 1 h of inactivity. In model 1, unique regression parameter was estimated, with different estimates for H1 and H2 over all modules. In model 2, H1 and H2 regression parameter values was constrained to be equal over all modules, one value for H1 and one for H2, respectively (41). Then a partially constrained model was tested with all constrained to be equal for either H1 or H2 and the other hypothesis freely estimated with unique parameter values. This procedure tested model parsimony in order to keep the model as simple as possible (42), which also kept statistical power at highest possible level. Continuous outcome measures were tested with linear models, while dichotomous and ordinal outcomes were estimated with logit models. Potential non-normality was handled with Maximum Likelihood Robust (MLR) in order to give correct standard errors (43). Full information maximum likelihood (FIML) use all available data and assumes missing data to be randomly distributed (MAR) (44). This is a less restrictive assumption than missing completely at random (MCAR). However, we cannot rule out the possibility of missing being not at random (MNAR), which is not a direct testable hypothesis.

### Ethics

The study was approved by the Norwegian Regional Committees for Medical and Health Research Ethics, Region West (90483). All participants signed an informed consent form. The trial was registered retrospectively on August 13th, 2020, on clinicaltrials.gov (NCT04511169).

## Results

### Participants

**Figure 2:**
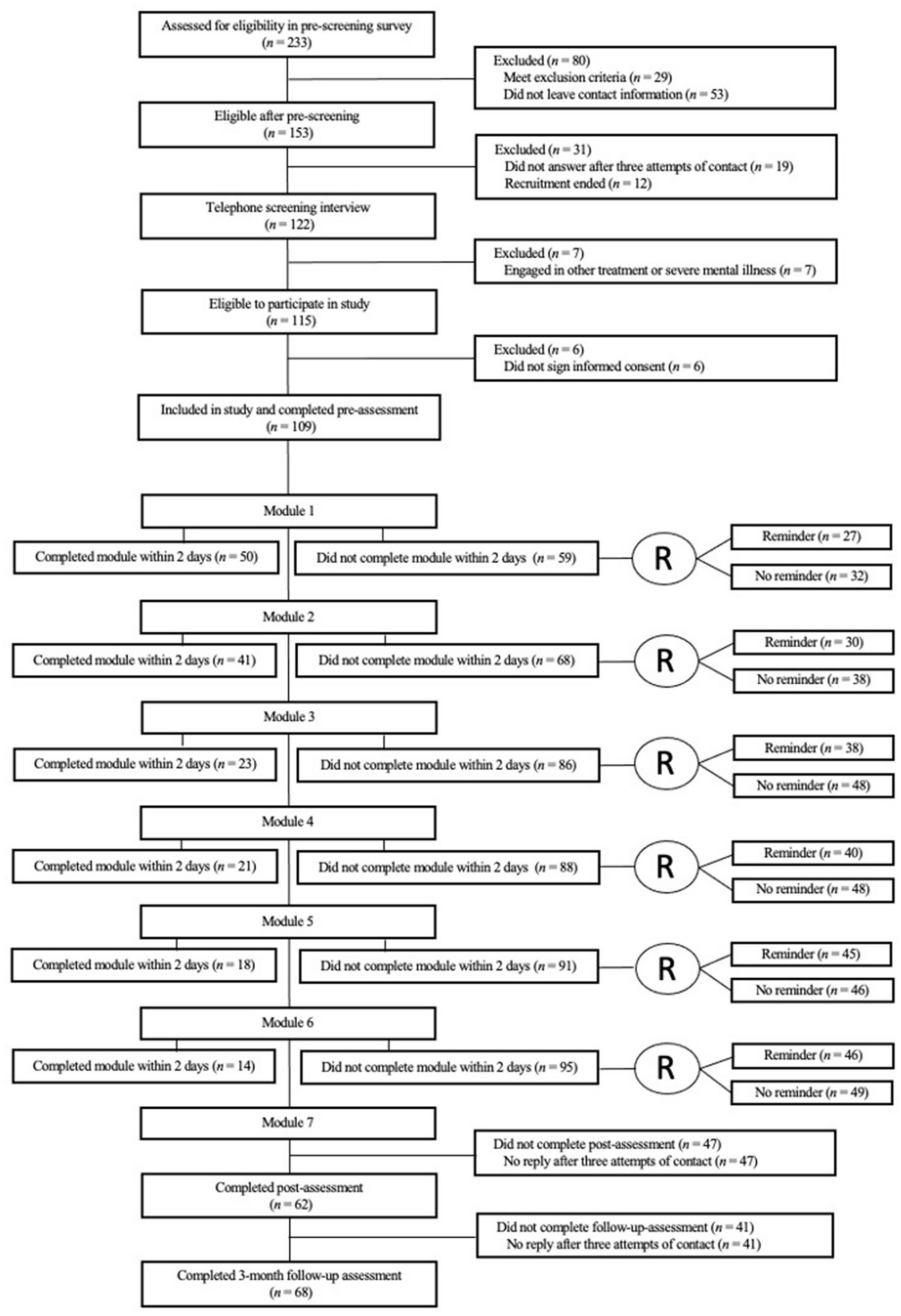
Study flowchart.

The recruitment started 8th of May 2020 and ended 30th of May 2020. During the recruitment phase, 233 accessed the online pre-screening survey, and 115 met the inclusion criteria. However, six participants never accessed the intervention portal to sign the informed consent, leaving the sample size at 109 (see Figure 2 for flowchart).

Among the 109 included participants, 80.7% were females (see Table 1 for demographics). All but five of the participants were diagnosed with ADHD in adulthood (i.e., after 18 years of age).

**Table 1:** Demographic characteristics of participants, *n* = 109.

	***n*/*M***	**%/*SD***
Gender		
Female	88	80.7%
Male	21	19.3%
Age	36.1	9.1
Age of diagnosis	30.5	9.2
Currently receives ADHD medication	86	78.9%
Employment		
Full-time employed or student	74	67.9%
Sick leave, disability pension or work allowance assessment	30	27.5%
Homemaker or unemployed	5	4.6%
Education		
Elementary school	11	10.1%
High school	36	33.0%
University or college	62	56.9%

*n, number of participants; M, mean; SD, standard deviation*.

### Adherence

See Table 2 for overview of adherence outcomes. A total of 56% of the participants (*n* = 61) completed at least five out of seven modules and were thus defined as treatment completers. The mean number of completed modules among the participants was 4.6 (SD = 2.6).

**Table 2:** Descriptives of adherence measures.

**Adherence measures**	***n***	***M***	***SD***	**Range**
Number of completed modules	109	4.6	2.6	0–7
Number of logins	109	9.3	7.1	2–47
Total minutes in the program^a^	108	180.5	148.1	9–783
Self-reported practice of coping techniques	61	4.0	1.3	1–6

a *One participant was excluded due to extreme scores*.

*n, number of participants; M, mean; SD, standard deviation*.

### The Effect of SMS Reminders

See Figure 2 for an overview of the participants included in the weekly randomization. Using Chi Square for investigation of between-group differences, SMS reminders were only associated with an increased likelihood of login within 48 h during the second week of the intervention (*p* = 0.049) while no significant effects were found for module completion (see Table 3). In the following we present the results of the main analyses for each of the adherence outcomes.

**Table 3:** Between-group differences in weekly module completion and logins.

**Module**	**Group**	***n***	**Login within 2 days after reminder** ***n* (%)**	**Module completion within a week** ***n* (%)**
2	Reminder	30	12 (40.0%)^*^	15 (50.0%)
	No reminder	38	7 (18.4%)	16 (42.1%)
3	Reminder	38	11 (28.9%)	12 (31.6%)
	No reminder	48	15 (31.2%)	17 (35.4%)
4	Reminder	40	7 (17.5%)	9 (22.5%)
	No reminder	48	7 (14.6%)	12 (25.0%)
5	Reminder	45	10 (22.2%)	10 (22.2%)
	No reminder	46	5 (10.9%)	8 (17.4%)
6	Reminder	46	7 (15.2%)	10 (21.7%)
	No reminder	49	7 (14.3%)	5 (10.2%)

*n, number of participants*;

* *P < 0.05 based on Chi-square analysis*.

#### Module Completion

The two tested hypotheses H1 and H2 with result constrained to be equal over all modules are shown in Table 4. With regards to module completion, the participants who were included in the randomization completed fewer modules than participants not included in the randomization across time (H1 hypothesis). In the unconstrained model, module completion was found to be related to the H1 contrast in module two and five (b = −1.74, *p* < 0.001 and b = −1.17, *p* = 0.008). In the partially constrained model this result was −1.05 (*p* < 0.001).

**Table 4:** Outcome variables predicted by being in the randomization group or not (hypothesis H1) and receiving SMS or not in the randomized group (hypothesis H2).

	**H1**		**H2**	
	***b***	***P***	***b***	***P***
Module completion	−1.02	<0.001	0.16	0.473
Number of logins	−3.15	<0.001	−0.34	0.407
Total minutes in the program	−51.93	<0.001	0.19	0.985
Days practiced within a week^a^	−0.33	0.011	0.10	0.669

a *Never, less than once a week, 1–2, 3–4, 5–6 days or daily*.

The SMS reminder did not have any effect on module completion in the constrained or unconstrained model comparing the two subgroups included in the randomization (H2 hypothesis). However, in the partially constrained model, the SMS reminder given in module five was found to be associated with completing one more module in total (H2: b = 1.03, *p* = 0.032).

#### Logins

Participants in the randomization group had a lower number of logins compared to the participants not included in the randomization (constrained model). The partially constrained model showed this estimate to be −3.18, *p* < 0.001. In the unconstrained model, this difference in logins was found in module three and five (b = −3.53, *p* = 0.024; b = −5.04, *p* = 0.015). No effect of SMS reminders was found on number of logins in the unconstrained, constrained or partially constrained models.

Examining the time to first login, the results from the partially constrained model (with module 3 and 5 to have equal magnitude in relation), showed that receiving an SMS reminder was associated with using 63.66 less minutes before the first login compared to not receiving a reminder (see Table 5). However, no effects of the SMS reminder on time to login were found in the other modules. The participants that were not included in the randomization to receive SMS were estimated to use 122.55 less minutes on time to login across the modules (*p* < 0.001). No Lag 1 and Lag 2 constrained relations were found (Lag 1: H1: b = 4.91, *p* = 0.789, H2: b = 33.94, *p* = 0.214; Lag 2: H1: b = −13.29, *p* = 0.486, H2: b = 24.72, *p* = 0.203), nor did the analyses show any unconstrained lag-relations to be statistically significant.

**Table 5:** Time to first login predicted and minutes in the modules by being in the randomization group or not (hypothesis H1) and receiving SMS or not in the randomized group (hypothesis H2).

	**Time to first login**				**Minutes in modules**			
	**H1**		**H2**		**H1**		**H2**	
	**b**	***P***	**B**	***P***	**b**	***P***	**b**	***P***
Module 2	111.51	<0.001	2.38	0.944	0.64	0.868	7.16	0.275
Module 3	93.44	<0.001	−68.22	0.046	−9.16	0.719	−46.00	0.568
Module 4	139.52	<0.001	31.68	0.340	−10.85	0.033	2.17	0.479
Module 5	169.80	<0.001	−60.95	0.055	−2.72	0.319	2.54	0.300
Module 6	119.95	<0.001	0.123	0.997	2.95	0.600	11.30	0.096

#### Minutes Spent Online

Total amount minutes spent on the program was found to be 52 min lower for the participants included in randomization for the SMS reminder compared to the other participants (constrained model). In the unconstrained model it was found that this difference was accounted for the group difference in module five (b = −116.73, *p* = 0.031). None of models showed any effect of the SMS reminder (constrained model: b = 0.19, *p* = 0.985; partially constrained model: b = −32.44 to 29.74, *p* > 0.179).

The number of minutes spent on each module showed that participants included in the randomization to receive an SMS reminder in module 4 spent less time on this module (b = −10.85, *p* = 0.033) (unconstrained model). In the constrained model, an effect for the SMS reminders was found, with the participants receiving an SMS reminder spending 3.62 more minutes in average over all modules compared to the in randomized group not receiving an SMS. In this model, no difference was found among those included in the randomization group and those not included in the randomization (b = −3.46, *p* = 0.117). In the partially constrained model, the randomization group used 11.18 less minutes in module 4 than those not included in the randomization (*p* = 0.035). This model also showed that the SMS group spent 3.94 more minutes on all modules compared to the randomized group not getting the SMS reminder (*p* = 0.037). No Lag 1 or Lag 2 relationships were found.

#### Practice of Coping Strategies

The self-reported average amount days a week spent on practicing the coping strategies, ranging from never to daily, was found to be related to H1 (b = −0.33, *p* = 0.011) in the constrained model. However, no effect of the SMS reminder was found on this adherence outcome (b = 0.10, *p* = 0.669).

## Discussion

This is the first study to examine the effectiveness of SMS reminders for adults with ADHD participating in a self-guided Internet-delivered intervention. The results showed that the participants who received an SMS reminder were more likely to login to the intervention within 48 h during the second module of the intervention, but no differences were found in any other modules. SMS reminders were associated with spending more time on the intervention modules and using less time to login into module three and five, specifically. However, the overall results did not show an effect of SMS reminders on module completion, number of logins or practice of coping strategies.

In terms of logins, it was found that SMS reminders were associated a higher likelihood of login in module two and faster login in modules three and five. We expected the SMS to have the strongest effect on login during the first modules of the intervention, however, the current results do not appear to be associated with time progressed in the intervention. The significant effects on time to login found in module 3 and 5, may be explained by the specific module content, where module 3 was centered around inhibition control and module 5 on organization and planning. These modules may have been viewed as more relevant to their daily life situations than the other modules. Still, no effect of SMS reminders was found on number of logins in any of the modules. As such, the results support that SMS reminders may lead to faster login in some modules, but this effect was not consistent across all modules.

When it comes to time spent on the program and intervention modules, it was found that receiving an SMS reminder was associated with spending more time on the intervention modules. However, when examining total minutes spent on the program, we did not find any significant effect of the SMS reminders. This may be explained by the procedure, where the pre, post and follow-up assessment were conducted as part of the program and thereby add to the total time measure.

With regards to module completion, the overall results did not show a significant effect of SMS reminders. This is in contrast to findings from previous research on self-guided Internet-delivered interventions, showing a significant effect of reminders on module completion rates (20). One reason for this disagreement may be related to sample differences. Previous studies have not included adults with ADHD, but patients with other psychiatric disorders, such as anxiety and depression (20). It may be that some of the characteristics of ADHD could make treatment adherence more challenging than in other patient groups (12). For instance, a study that examined the effect of SMS reminders on treatment adherence among patients with alcohol dependence found that those with higher levels of impulsivity benefitted less from SMS reminders (45). However, Biederman et al. (31) found that SMS reminders improved adherence to pharmacological treatment among adults with ADHD, indicating that SMS reminders may be effective for this patient group as well. Still, it is important to highlight that there are differences between pharmacological and psychological interventions for ADHD, where the mechanisms underlying non-adherence in psychological interventions may be different, and perhaps more complex, than those involved in pharmacological interventions. As such, it is possible that SMS reminders do not target the specific barriers to adherence in psychological interventions for adults with ADHD.

The design and format of the SMS reminders should also be considered when interpreting the findings. Firstly, the SMS reminders in the current study were only sent once per week, and it is possible that this frequency was not sufficient (46). Still, it is not evident that more frequent reminders would have been more effective, as participants could find multiple reminders to be intrusive or bothersome, and thereby compromise treatment satisfaction (47). Secondly, the participants were not able to decide the time of day they wished to receive the SMS reminders. Reminders has been suggested to be most effective when given at convenient times (47, 48). Hence, it is possible that the participants would have benefitted more from the SMS reminders if both the time of delivery and frequency of the reminders were decided by themselves. Thirdly, the SMS reminders only included a generic message reminding the participants to continue with the program. It has been suggested that tailoring reminders to make them more relevant to the participants' own situation or experiences is more effective than generic reminders (46, 49). Perhaps it would have been of greater benefit to the participants if the SMS reminders had included more personalized content, such as positive reinforcement about progress already made, or more motivational content, such as information about the advantages of treatment adherence. Lastly, all participants received an SMS when a new module was available, consequently, the randomized SMS reminder was not the only prompt given to the participants throughout the intervention. Therefore, it may be that this first SMS created a ceiling effect, reducing the effect of the randomized SMS reminder. As such, the results from the study indicate that an additional SMS reminder beyond a SMS notifying participants about new modules does not add an extra benefit on module completion, login rates or use of coping strategies.

## Strengths and Limitations

With 109 participants, this study is one of the largest studies to investigate Internet-delivered interventions for adults with ADHD, and the first to examine the effect of SMS reminders on adherence to psychological treatment among adults with ADHD. Some limitations should be considered. Almost 40% of the participants did not complete the post- and follow-up assessment, and most of the included participants were females. In addition, the SMS reminder represents a minor intervention and large effects could thus not be expected to be found, especially since all participants received SMS reminders when a new module was available. Another limitation is that we did not use a-priori power analysis. During the first week of the trial there was also a technical error causing the SMS reminders to be trigged by 1 h of inactivity. This first-week module was therefore excluded from all statistical analyses, preventing conclusions about an early effect of reminders. Moreover, the study started 2 months into the COVID-19 pandemic, with the follow-up assessment conducted 7 months into the pandemic. This may of course have influenced adherence rates.

## Conclusions

The results from the study indicate that SMS reminders do not have an effect on module completion, login rates, or practice of coping strategies in a self-guided Internet-delivered intervention for adults with ADHD. However, SMS reminders may lead to faster login time and more time spent on the intervention modules. The discrepancy in findings across the outcome measures could point to the value of including several measures on adherence to get an improved picture on adherence in Internet-delivered interventions. Future research should investigate whether the use of tailored reminders, motivational reminders, or more novel approaches, such as gamification, can produce stronger effects on adherence rates in self-guided interventions for adults with ADHD.

## Data Availability Statement

The raw data supporting the conclusions of this article will be made available by the authors, without undue reservation.

## Ethics Statement

The studies involving human participants were reviewed and approved by the Norwegian Regional Committees for Medical and Health Research Ethics, Region West. The patients/participants provided their written informed consent to participate in this study.

## Author Contributions

TN is the head of the INTROMAT project. AL is the domain expert for the ADHD subproject. TN, EN, and AL contributed to the recruitment and data collection. RG was responsible for the statistical analyses and interpretation of results. EN drafted the manuscript. TN, RG, AL RK, FG, and SM critically reviewed the manuscript. All authors contributed with the conception and study design and approved the final version of the manuscript.

## Funding

This study is part of the INTROMAT project, funded by the Research Council of Norway (grant: 259293). EN received funding from the Western Norway Regional Health Authorities (Helse Vest) for her doctoral thesis.

## Conflict of Interest

The authors declare that the research was conducted in the absence of any commercial or financial relationships that could be construed as a potential conflict of interest.

## Publisher's Note

All claims expressed in this article are solely those of the authors and do not necessarily represent those of their affiliated organizations, or those of the publisher, the editors and the reviewers. Any product that may be evaluated in this article, or claim that may be made by its manufacturer, is not guaranteed or endorsed by the publisher.
